# Adaptation and Validation of the ADOS-2, Polish Version

**DOI:** 10.3389/fpsyg.2017.01916

**Published:** 2017-11-07

**Authors:** Izabela Chojnicka, Ewa Pisula

**Affiliations:** Faculty of Psychology, University of Warsaw, Warsaw, Poland

**Keywords:** autism spectrum disorders, diagnosis, Autism Diagnostic Observation Schedule, ADOS-2, adaptation, validation

## Abstract

Autism Diagnostic Observation Schedule (ADOS) is one of the most popular instruments used world-widely in the diagnosis of autism spectrum disorders (ASD). Unfortunately, there are only a few studies of the psychometric properties of non-English language versions of this instrument and none of the adaptation of its second edition (ADOS-2). The objective of this study was to verify the psychometric properties of the Polish version of the Autism Diagnostic Observation Schedule, Second Edition (ADOS-2-PL). The authors recruited 401 participants: 193 with ASDs (ASD group) and 78 with non-spectrum disorders, plus 130 typically developing participants (control group). ADOS-2-PL was found to have high interrater reliability, internal consistency and test–retest reliability. Confirmatory factor analysis confirmed a good fit of the Polish data to the two-factor model of ADOS-2. As no significant differences were found between participants with childhood autism and other ASDs, only one cut-off was established for Modules 1–4. The sensitivity, specificity and positive predictive value of ADOS-2-PL are high: sensitivity was over 90% (only for the “Older with some words” algorithm in the Toddler Module the sensitivity was 71% and “Aged 5 years or older” algorithm in Module 2 sensitivity was 84%), specificity was above 80% (with the exception of the Module 4 and Module 2 “Aged 5 years or older” algorithm where it was above 70%). The results support the use of ADOS-2-PL in clinical practice and scientific research. To the best of our knowledge, there have been no reports to date about adaptations of ADOS-2 and the psychometric properties of non-English language versions. As such, this constitutes the first attempt at adapting ADOS-2, and its results could be of interest for researchers outside of Poland.

## Introduction

Autism spectrum disorders (ASD) are a heterogeneous group of neurodevelopmental disorders that continue to pose considerable diagnostic challenges to professionals and researchers (Jeste and Geschwind, 2014). The validity and reliability of ASD diagnosis improves significantly when it is based on a comprehensive diagnostic evaluation, using standardized instruments with good psychometric properties (Kim and Lord, 2012; Guthrie et al., 2013). The most popular of those instruments is the *Autism Diagnostic Observation Schedule* (ADOS, Lord et al., 1999), which, along with the *Autism Diagnostic Interview-Revised* (ADI-R, Rutter et al., 2003b), is considered the gold standard in ASD diagnosis (Falkmer et al., 2013).

Autism Diagnostic Observation Schedule is a semi-structured, standardized assessment of social interactions, language and communication, repetitive, restricted patterns of behavior and interest, as well as play and imagination (Lord et al., 1999). ADOS contains four modules differentiated by participant’s developmental and language levels, ranging from Module 1 for non-verbal individuals with non-verbal mental age of at least 15 months to Module 4 for verbally fluent older adolescents and adults. Every ADOS module ends with a diagnostic algorithm(s) that consists of selected items that have been chosen to maximize diagnostic sensitivity and specificity. The instrument allows for direct observation of the behavior exhibited by individuals referred for a possible ASD. As such, ADOS supplements clinical diagnosis by providing objectivized information related to ASD diagnostic criteria (World Health Organization [WHO], 2002; American Psychiatric Association [APA], 2013). It is also used to obtain information useful in treatment planning and educational placement. ADOS has become a standard in research studies, ensuring standardized inclusion criteria in terms of the presence and severity of symptoms typical for ASD and reliability of comparisons of results obtained in different research centers, making them also more readily comparable.

*Autism Diagnostic Observation Schedule, Second Edition (ADOS-2)* is an updated and expanded version of ADOS (Lord et al., 2012a,b). It contains five assessment modules for testing individuals of different chronological ages and at different developmental levels. The structure of the ADOS-2 Protocol Booklets is presented in **Table 1**. The existing ADOS Modules 1–4 were expanded with the Toddler Module, designed specifically for young children aged 12–30 months with limited expressive language. The ADOS-2 enhances the original measure with revised algorithms for greater sensitivity and specificity. These additions have significantly increased the versatility of the instrument in early diagnosis of ASD and in monitoring the development of children at risk for autism as early as in the second year of life.

**Table 1:** Structure of an ADOS-2 Protocol Booklet.

**Administration section**			
Order and structure of the administration			
Observation and administration guidelines			
**Coding section**			
Items listed by five subsections: Language and Communication; Reciprocal Social Interaction; Play, Stereotyped Behaviors and Restricted Interests; Other Abnormal Behaviors.			
Examiner chooses one rating of the following: 0, 1, 2, 3, 4, 7, 8, 9 accordingly to Coding Conventions described in the manual and specific descriptions in a particular item.			
**Algorithm form**			
Each Module contains one or two diagnostic algorithms:			
Toddler Module Algorithms	• All younger/older with few to no words – for children with chronological age between 12 and 20 months OR children aged 21–30 months who used fewer than five words during an ADOS-2 assessment.		
	• Older with some words – for children with chronological age between 21 and 30 months who used at least five different words during an ADOS-2 assessment.		
Module 1 Algorithms	Fewer than five words – for children who used fewer than five words during an ADOS-2 assessment.		
	Five or more words – for children who used at least five different words during an ADOS-2 assessment.		
Module 2 Algorithms	Younger than 5 years – for children with chronological age below 5 years 0 months.		
	5 years or older – for children with chronological age at least 5 years 0 months.		
Module 3 Algorithm	For all participants assessed with Module 3.		
Module 4 Algorithm	For all participants assessed with Module 4.		
Examiner converts item codes to algorithm scores:			
• ratings of 3 to algorithm scores of 2			
• ratings of 7, 8, 9 to algorithm scores of 0			
Examiner does not convert ratings of 0, 1, and 2.			
Examiner transfers ratings of 0, 1 and 2 directly to the algorithm scores into two domains: Social Affect (SA) and Restricted and Repetitive Behavior (RRB).	Sum of scores within SA domain = SA Total Sum of scores within RRB domain = RRB Total	SA Total + RRB Total = Overall Total	Examiner coverts the Overall Total to the *ADOS-2 Classification* (two cutoffs: for *autism* and lower for *autism spectrum*) and the *ADOS-2 Comparison Score* (10-point severity metric).

*Based on Lord et al. (2012a,b).*

Both ADOS and ADOS-2 have good psychometric properties (Lord et al., 1999, 2012a,b). They are characterized by high interrater and test–retest reliability, as well as high validity, confirming their usefulness in distinguishing individuals with ASD from other clinical groups (e.g., Mazefsky and Oswald, 2006). The instrument is continually being developed to improve its diagnostic validity, with new standardized severity scores and revised algorithms recently added (Gotham et al., 2007, 2009; Hus and Lord, 2014; Esler et al., 2015). The algorithms were revised to be more comparable across modules [added Calibrated Severity Scores (CSS) – a standardized version of ADOS-2 scores that is less strongly associated with age and language compared to raw ADOS-2 totals] and better reflect ASD core symptoms (new algorithms include scores in both Social communication and Restricted, repetitive behaviors domains whereas previous algorithms did not take into account scores in Restricted and repetitive behaviors domain). Introduced CSS can serve as a symptom severity measure and may be valuable for comparing ADOS assessments across time and modules (de Bildt et al., 2011).

Most research on the psychometric properties of ADOS and ADOS-2 to date has been conducted in the US, using the original, English version of the assessment. Relatively little is known about its adaptations and the properties of other language versions.

A handful of studies on the reliability and diagnostic validity of other language versions of ADOS suggest that they retain the good psychometric properties of the original. The German language version features good interrater and test–retest reliability and fair internal consistency (Bölte and Poustka, 2004). The interrater agreement between this ADOS version and ADI-R is 79 and 77% with clinical diagnosis. As for discriminating between autism and other autistic disorders, the sensitivity was 90.4%, and its specificity was 48.1% in the German study. A slightly better specificity was reported by Papanikolaou et al. (2009), who investigated the interrater agreement between the Autism Diagnostic Observation Schedule (Lord et al., 2000) and the clinical diagnosis of autism in a Greek sample. In this study, interrater agreement was calculated for two variants: dichotomous (autistic disorder and PDD-NOS vs. non-ASDs) and trichotomous diagnosis (autistic disorder vs. PDD-NOS vs. non-ASDs). In both variants the interrater agreement of ADOS with clinical diagnosis was moderate to satisfactory for the Modules 1-3 evaluated in the study. The interrater agreement between ADOS and ADI-R varied across modules, ranging from the highest value for Module 1 to statistically non-significant for Module 3. Authors explained that obtained results were in accordance with previous research showing that concordance between the two instruments is greater in younger children (de Bildt et al., 2004). The ADI-R algorithm scores rely mainly on the 4–5 age period while the ADOS algorithm on current behavior. Symptoms might have become more subtle as children, especially those without intellectual disability, get older and receive intervention. Secondly, ADI-R and ADOS-2 use different source of information, the first one relies on parent-report, the latter on behavior observation. The two tools, ADI-R and ADOS, are recommended to be used together as they display the highest diagnostic validity, particularly when used in combination (Falkmer et al., 2013).

A series of studies on the properties of ADOS conducted in large Dutch samples tested the diagnostic validity of the original ADOS scoring algorithms (Lord et al., 1999) and of the revised algorithms proposed by Gotham et al. (2007), as well as verified the factor structure of ADOS (de Bildt et al., 2009, 2011; Oosterling et al., 2010). The studies supported the validity of collapsing two of the original ADOS domains – Social and Communication – into a single factor, namely Social Affect (SA). The ADOS algorithms revised by Gotham et al. (2007) were also tested in a study on a very small sample of children in Spain (Overton et al., 2008). The findings varied depending on the module, with the best values obtained for Module 1 and in the more severe autistic group.

Due to its good psychometric properties coupled with clinical merits, ADOS has been established as the first-choice diagnostic tool in ASD in United States, Europe and other parts of the world (Zander et al., 2015). In Poland the need for standardized diagnostic tools for ASD is still unmet. One of the reasons for developing the Polish version of ADOS-2 was to help fill this gap.

There has been some progress in understanding the symptoms of ASD and access to diagnosis and treatment of autism in Poland recent years. This view is supported by a cross-cultural study indicating similarity in perception of early ASD symptoms in Poland, Greece, Italy, Japan and United States (Matson et al., 2017). Although Polish diagnostic centers usually follow best practice guidelines of a comprehensive diagnostic evaluation for autism (National Autism Center, 2015), since validated Polish versions of standardized instruments to be used in ASD diagnosis are lacking, each diagnostic center uses its own interview protocols and unstructured observation (Pisula, 2012). The assessment is based on the International Classification of Diseases-Tenth Edition (ICD-10 diagnostic criteria; World Health Organization [WHO], 2002), and, as required by the Polish law, should be made by a licensed psychiatrist or neurologist. The diagnostic process usually involves psychologists and other professionals, and includes an interview with a parent/caregiver of the diagnosed individual along with observational assessments (note: report in preparation). However, the methods used are not standardized, and their clinical value has not been empirically verified (Rozetti, 2015).

Lack of standard diagnostic procedures and instruments significantly undermines the reliability of diagnoses. This, in turn, complicates support for individuals with ASD and hampers empirical research on the Polish samples (Chojnicka and Ploski, 2012).

This article discusses the results of the project whose goal was to adapt ADOS-2 for use in Poland and to determine the reliability and validity of the Polish version of the instrument. The use of diagnostic tools developed for different cultural contexts requires careful adaptation, including tests of reliability and validity, as well as recalculation of cut-offs and standardized scores whose values may be different from the original (Hambleton et al., 2009). To the best of our knowledge, there have been no reports to date about adaptations of ADOS-2 and the psychometric properties of non-English language versions. As such, this constitutes the first attempt at adapting ADOS-2, and its results could be of interest for researchers outside of Poland.

## Methods

### Participants

There were 401 participants in the study, including 193 with autism spectrum disorders (ASD group) and 208 in the control group consisting of individuals with non-spectrum disorders (*N* = 78) and typically developing individuals (*N* = 130). Of the ASD group, 58.5% were diagnosed with childhood autism and 41.5% with other pervasive developmental disorders (Non-autism ASD, mostly pervasive developmental disorder unspecified or Asperger syndrome, as well as seven individuals diagnosed with atypical autism). All participants in that group have received a clinical diagnosis of ASD from psychiatrists who evaluated them using the ICD-10 diagnostic criteria (World Health Organization [WHO], 2002). In the Non-spectrum disorders group the majority were individuals with an intellectual disability or disorders of speech and language. Individuals with significant hearing, visual or motor impairments, as well as non-Polish speakers were disqualified from the study. As in the manual for the original version of ADOS-2 (Lord et al., 2012a,b), we avoided including individuals with ADHD, anxiety disorders and depression in the validation sample.

The study groups were structurally varied in terms of chronological age, sex and language level in a manner appropriate for the specifics of individual modules of ADOS-2 (**Table 2**). Among participants diagnosed with autism 85% males were included in the study, where a slightly lower percentage (80–83% of males) has been found to be typical in the ASD population (Christensen et al., 2016). The Non-autism ASD group included 21 females and 59 males, and the control group included 76 females and 132 males. In the whole sample (*N* = 401), 71% of participants were from cities with a population of 100,000 or more (from 66 to 82% in a given group), 19% (12–23%) were from towns with up to 100,000 inhabitants, and 8.5% (3–10%) were from rural communities. In 1.5% of cases information on the place of residence was missing.

**Table 2:** Characteristics of participants in ADOS-2-PL sample.

Toddler Module				
	Autism	Non-autism ASD	ASD group	Non-spectrum group
*N* (females, males)	24 (5, 19)	10 (4, 6)	34 (9, 25)	42 (14, 28)
Chronological age *M* (*SD*)	24.13 (4.6)	23.79 (4.9)	24.03 (4.48)	23.54 (3.96)
Age range (in months)	15–33	16–30	15–33	13–31
DSR *M* (*SD*)	82.16 (33.43)	104.0 (39.11)	90.35 (36.76)	101.63 (26.99)

**Module 1**				

	Autism	Non-autism ASD	ASD group	Non-spectrum group
*N* (females, males)	30 (6, 24)	11 (5, 6)	41 (11, 30)	40 (17, 23)
Chronological age *M* (*SD*)	54.7 (19.44)	42.91 (18.66)	51.53 (18.59)	42.53 (18.84)
Age range (in months)	30–103	32–45	30–103	32–164
Leiter Scale score *M* (*SD*)	79.0 (26.49)	64.86 (33.48)	75.70 (28.31)	86.42 (24.20)

**Module 2**				

	Autism	Non-autism ASD	ASD group	Non-spectrum group
*N* (females, males)	17 (3, 14)	23 (4, 19)	40 (7, 33)	41 (15, 26)
Chronological age *M* (*SD*)	72.81 (18.6)	55.77 (8.99)	62.97 (13.72)	69.97 (20.99)
Age range (in months)	39–148	35–92	35–148	24–107
Leiter Scale score *M* (*SD*)	91.11 (13.06)	106.0 (32.24)	101.41 (28.33)	114.2 (22.72)

**Module 3**				

	Autism	Non-autism ASD	ASD group	Non-spectrum group
*N* (females, males)	18 (1, 17)	21 (4, 17)	39 (5, 34)	43 (14, 29)
Chronological age *M* (*SD*)	139.83 (40.43)	115.71 (25.88)	126.85 (35.10)	119.79 (34.21)
Age range (in months)	79–233	82–171	79–233	68-188
WISC-R Verbal IQ *M* (*SD*)	76.08 (21.69)	103.82 (19.40)	95.55 (26.61)	97.40 (28.06)
WISC-R Nonverbal IQ *M* (*SD*)	85.50 (22.54)	107.35 (21.49)	96.24 (22.34)	103.29 (26.57)

**Module 4**				

	Autism	Non-autism ASD	ASD group	Non-spectrum group
*N* (females, males)	24 (2, 22)	15 (4, 11)	39 (6, 33)	42 (16, 26)
Chronological age *M* (*SD*)	19.97 (4.48)	19.22 (3.04)	19.73 (6.99)	22.89 (6.99)
Age range (in years)	15-35.4	16.3-27.4	15-27.4	15.9-42.3
WAIS-R Verbal IQ *M* (*SD*)	98.6 (16.47)	115.5 (10.61)	103.43 (16.36)	83.71 (27.24)
WAIS-R Nonverbal IQ *M* (*SD*)	99.40 (33.86)	95.0 (7.07)	98.14 (27.88)	83.57 (35.41)

*DSR, Children Development Scale, for DSR mean (M) and standard deviation (SD) are given for the overall score; WISC-R, The Wechsler Intelligence Scale for Children - Revised; WAIS-R, The Wechsler Adult Intelligence Scale - Revised.*

The majority of participants with ASD (∼60%) were individuals referred for ASD evaluation at diagnostic centers. Approximately 30% were diagnosed with ASD within 1 year prior to study enrolment. In the remaining cases (mostly participants assessed with Module 4), the time from diagnosis exceeded 12 months, and the participants’ current functioning was assessed by a psychiatrist based on ICD-10 diagnostic criteria (World Health Organization [WHO], 2002).

### Instruments and Procedure

#### Adaption of the ADOS-2 to the Polish Cultural Context

The translation of ADOS-2 into Polish consisted of several steps. Firstly, authors in cooperation with professional translator translated ADOS-2 from English into Polish. Secondly, the translation was subjected to proofreading by a native Polish linguist, correction and revision. Subsequently, the blind, back-translation was prepared by an independent, translation company. The back-translation was submitted for the ADOS-2 author review. Eventually, the translation was amended accordingly to authors’ comments and approved for the research use by the publisher, Western Psychological Services (WPS).

The Polish version of ADOS-2 (ADOS-2-PL) is very similar to the original. ADOS-2 translation into Polish preserves the structural equivalence associated with the graphical form of protocols, text and format of items, maintains translation accuracy including the contents of items, grammatical structure of questions, difficulty of terminology and lexical similarity of questions (Hambleton et al., 2009). Untranslatable expressions were replaced by Polish equivalents, which sometimes required the use of narrative descriptions or providing illustrative examples.

The activities that make up each of the five ADOS-2 modules and the stimulus items used are the same in Polish version. The only modification involves the choice of Polish songs sang during Bath Time in the Toddler Module and Birthday Party in Module 1.

The items constituting the ADOS-2 Coding sections in all five modules are the same in the Polish version. However, there was one issue needed to be addressed – a part of the A1a. “Frequency of Babbling” item from Toddler Module where the list of sounds that the child may use is provided: *m, n, b| p, d| t, g| k, w, l, y, s, sh*. The examiner is supposed to circle all sounds used by the child during ADOS-2 assessment. In cooperation with speech and language therapists, a few sounds were adapted in the Polish version. That is:

-‘*w*’ from original version was changed into Polish equivalent ‘*ł*’;
-‘*y*’ which is a special sound in English and can be regarded as both a vowel and a consonant. Since it was the only vowel in the list we decided to adapt it using its consonant sound (as in the words ‘*yellow’* or ‘*yogurt’*) and changed ‘*y*’ into Polish equivalent ‘*j*’(consonant);
-‘*sh*’ [∫] which lays somewhere in between Polish sounds ‘*ś*’ and ‘*sz*’. We do not expect a child to be using proper Polish ‘*sz*’ sound before 5 birthday (Czaplewska and Milewski, 2012), therefore we decided to use ‘*ś*’ sound in the Polish version which is used by children 12-18 months of age.

Based on the confirmatory factor analysis results (details in the Section Results), it was decided to retain the original diagnostic algorithms in the Polish version of ADOS-2. That means, that diagnostic algorithms in the ADOS-2-PL contain same items as the original ADOS-2. At the time, WPS Module 4 Protocol Booklet contained the algorithm before revision. Therefore, in the Polish version of Module 4 we used the revised algorithm developed and made available to the authors by the Center for Autism and the Developing Brain at the New York-Presbyterian Hospital, United States (Hus and Lord, 2014). For all eight of the ADOS-2 algorithms, the revised algorithms were used to compute SA and Restricted and Repetitive Behavior (RRB) domain totals and overall totals. In the ADOS-2-PL the calculation method of domain totals and overall totals is the same as in original algorithms: the ratings assigned during coding for the algorithm items are converted to algorithm scores (assigned ratings of 3 should be converted to algorithm scores of 2; and assigned ratings of 7, 8, and 9 should be converted into scores of zero), which are summed to receive domain totals and overall totals. Overall totals are compared with the cutoffs to determine ADOS-2 Classification. Instead of the two cut-offs (separate for *autism* and *autism spectrum*), as in the original ADOS-2 Classifications (Lord et al., 2012a,b), only one cut-off was determined for the entire autism spectrum in ADOS-2-PL (details in the Section Results). The exception was the Toddler Module, which is scored on a three-point ADOS-2 Range of Concern scale just as the original one.

#### Other Instruments

##### Autism Diagnostic Interview-Revised

For the convergent validity analysis we have used author-reviewed Polish version of the Autism Diagnostic Interview-Revised (ADI-R) accepted for the research use by the Western Psychological Services, the copyright holder. ADI-R is a comprehensive, standardized, semi-structured interview useful for diagnosing autism, planning treatment, and distinguishing autism from other developmental disorders in children and adults with a mental age above 24 months (Rutter et al., 2003b). ADI-R may be used also for assessing younger children using recently published new algorithms for toddlers and young preschoolers from 12 months of age (de Bildt et al., 2015).

The ADI-R is a complex protocol composed of 93 items in three main domains of functioning – language/communication; reciprocal social interactions, social development and play; and restricted, repetitive, and stereotyped behaviors and interests. ADI-R assesses also other aspects of behavior such as the subject’s background; overview of the subject’s behavior; early development and developmental milestones; language acquisition and loss of language or other skills; other clinically relevant behaviors like aggression, self-injury, and possible epileptic seizures.

Polish version of ADI-R is characterized by good psychometric properties confirming its usefulness both in individual clinical diagnostics of ASDs and in scientific research (Chojnicka and Pisula, 2017).

##### Social Communication Questionnaire

Another tool used for the convergent validity analysis was author-reviewed Polish version of the Social Communication Questionnaire (SCQ), accepted for the research use by the Western Psychological Services, the copyright holder. SCQ is a parent questionnaire useful for screening purposes in children over 4.0 years, with a mental age over 2.0 years who may have ASDs (Rutter et al., 2003a). It consists of 40 yes-or-no items concerning communication skills and social functioning. Similarly, to ADI-R, Polish version of SCQ is characterized by good psychometric properties (Pisula et al., 2017, Unpublished manuscript).

##### Child Development Scale

To assess the developmental level of the youngest participants we have used the Polish Child Development Scale (Dziecięca Skala Rozwojowa, DSR, Matczak et al., 2007). DSR is a comprehensive standard measurement to assess the development of children from 2 to 36 months of age. It provides assessment of cognitive development, fine motor and gross motor development, auditory and visual perception, memory, receptive and expressive language, social behaviors and play. It is a useful, characterized by good psychometric properties, instrument for screening purposes, determining child’s strengths and difficulties or intervention planning.

##### Leiter International Performance Scale

Cognitive abilities of nonverbal participants aged 3.0–15.11 were assessed using Leiter International Performance Scale (LEITER; Polish adaptation by Jaworowska et al., 2009). LEITER is a completely nonverbal measure of intelligence recommended for children with autism spectrum diagnosis. Polish adaptation is characterized by good psychometric properties. It assesses fluid intelligence, considered by many the truest measure of a person’s innate ability. The LEITER IQ score reflects participant’s cognitive abilities is not significantly influenced by her/his language skills, or by educational or social experience.

##### Wechsler Intelligence Scales

Intellectual abilities of verbal participants were assessed using Wechsler Intelligence Scales.

Children and adolescents aged 6.0–16.11 were assessed using Wechsler Intelligence Scale for Children – Revised (WISC-R, Polish adaptation by Matczak et al., 2008). Verbal participants older than 16.11 were assessed Wechsler Adult Intelligence Scale (WAIS-R, Polish adaptation by Brzeziński et al., 2004). Polish adaptations of Wechsler Intelligence Scales are characterized by good psychometric properties and consist of Verbal and Performance scales and provide scores for Verbal IQ, Performance IQ, and Full Scale IQ.

#### Procedure

The ADOS-2-PL was conducted as part of a research evaluation. Each project participant was rated independently by two professionals trained in the use of the instrument for scientific and clinical purposes. Almost half (45%) of assessments were conducted “live” (in the presence of two experimenters, one of whom was the observer). In the remaining cases, one examiner assessed a participant’s behavior live, and one from a video recording. In 20% of cases the assessment was performed independently by an examiner working at a different diagnostic facility than the diagnostician conducting the ADOS-2 assessment. ADOS-2 was conducted by 16 professionals, among them psychologists, educators, speech therapists, and a psychiatrist. Each examiner established research reliability on the ADOS-2 and achieved at least 80% agreement for ADOS-2 scores. This part of the project was supervised by an independent ADOS-2 trainer, who trained the examiners performing assessments with ADOS-2 and looked after maintenance of examiners’ reliability in the course of the project. During the project, the ADOS-2 trainer re-established her own interrater reliability with independent, international ADOS-2 trainers.

Almost half of the participants were reassessed in order to estimate the stability of ADOS-2-PL scores. Thirty-six participants were retested using the Toddler Module, along with 37, 38, 35, and 28 participants reassessed using Modules 1-4, respectively. Times to retest were as follows: 1–3 months for the Toddler Module (mean interval between tests = 2 months), 1–7 months for Module 1 (mean interval 4 months), 1-9 months for Module 2 (mean interval 5 months), 1-10 months for Module 3 (mean interval 6 months), 1–12 months for Module 4 (mean interval 6 months). In the original ADOS-2 reliability sample participants were retested within an average of 10 months for Modules 1–4 (Lord et al., 2012b) and within 2 months for Toddler Module (Lord et al., 2012a). So, timing of retesting was similar to that described in the original ADOS-2 manual (Lord et al., 2012a,b).

In order to obtain additional information about ASD symptoms presented by the participants, some of them were tested with the Polish author-reviewed version of the ADI-R (*N* = 120, ∼30% of sample) and with the author-reviewed Polish version of the Social Communication Questionnaire (SCQ, *N* = 240, ∼60% of sample).

Cognitive abilities were assessed using four instruments appropriate to the participants’ age and language abilities: the DSR used for 25% of the sample; Leiter International Performance Scale used for 35% of the sample; WISC-R used for 24% of the sample; WAIS-R used for 16% of the sample.

The assessments were conducted in four cities in Poland. Participants were contacted through diagnostic and therapeutic centers specialized in diagnosing autism spectrum and other disorders, as well as foundations and associations supporting individuals with developmental disabilities, nurseries, kindergartens and various educational institutions.

The project was approved by the Faculty’s of Psychology, University of Warsaw Research Ethics Committee (Address: Stawki 5/7, 00-183 Warsaw, Poland). The parents of participating children under 16 years of age signed informed consent forms prior to participation in the study. In the case of participants aged 16 and older, informed consents were signed both by the parent and participant. Each assessment was video-recorded with the consent of participants and/or their parents or caregivers. Recording was also approved by the Ethics Committee.

### Design and Analysis

In order to verify the usefulness of the ADOS-2-PL in the diagnosis of ASD in Poland and replicate its validity in an independent sample statistical analyses used to assess reliability and validity of ADOS-2-PL followed a procedure similar to the one described in the original ADOS-2 manual (Lord et al., 2012a,b).

The sample was divided by age and language level within each module to yield the eight developmental cells, i.e., eight ADOS-2 diagnostic algorithms: Toddler Module, All younger/older with few to no words cell; Toddler Module, Older with some words cell; Module 1, Fewer than five words cell; Module 1, Five or more words cell; Module 2, Younger than 5 years cell; Module 2, 5 years or older cell; Module 3 and Module 4). For each participant, domain and overall totals were calculated by adding algorithm item scores appropriate to the developmental cell. For reliability analyses, scores of 7, 8, and 9 were converted to zeros, while scores of 3 were recoded to 2, as they appear on the algorithm.

The reliability of ADOS-2-PL was estimated using three methods: interrater reliability (percent agreement, weighted kappas, and intraclass correlation coefficients), test–retest reliability (intraclass correlations) and internal consistency reliability (by calculating Cronbach’s alpha). Computations were done using IBM SPSS Statistics 17.0 suite (Spss Inc. Released, 2008).

In order to verify the factor structure of ADOS-2, confirmatory factor analysis (CFA) was conducted, checking the fit of data obtained in each ADOS-2 module to the original two-factor models (with two distinct domains: SA and RRB). The analysis used the maximum-likelihood estimation method. Calculations were done using the SPSS Amos 17.0 suite (Arbuckle, 2008). Unlike in the reliability estimations, in these analyses the scores of 7, 8, and 9 were marked as missing values and excluded. Information conveyed by those scores is radically different in terms of content from information coded as “zero.” We decided that taking them into account would distort the actual structure of algorithms in the factor analysis (for instance, a score of 8 in the item *Stereotyped/Idiosyncratic Use of Words or Phrases* means that the participant’s language was too limited to judge, whereas a score of 0 indicates absence of stereotyped/idiosyncratic language together with some spontaneous, non-echoed expressive language).

To determine the ADOS-2-PL discriminant validity, we compared the scores obtained in ADOS-2-PL domains and Overall Totals of Toddler Module and Modules 1–4 by Autism, Non-autism spectrum and Control groups, a one-way ANOVA was conducted, followed by tests of contrasts. The test of contrasts for independent samples was also employed to compare pooled Autism and Non-autism spectrum group (referred to as ASD spectrum) with controls.

To confirm the diagnostic validity of ADOS-2-PL we evaluated the sensitivity and specificity by applying Receiving Operating Characteristics (ROC) curves. In this analysis as well, scores of 3 were recoded to 2, while scores of 7, 8, and 9 were converted to zeros.

Cohen’s kappa (κ) was used to detect pair-wise agreement between ADOS-2-PL diagnosis and clinical diagnosis, as well as ADI-R diagnosis and SCQ diagnosis to verify convergent validity. In addition, logistic regression was done to evaluate the impact of age, sex, and cognitive level on the ADOS-2-PL diagnosis.

## Results

### Interrater Reliability

Interrater agreement for Overall Total scores and SA and RRB domain scores was measured using interclass correlation coefficients (ICCs). High ICC values were obtained for all algorithms (**Table 3**).

**Table 3:** Intraclass Correlations for Interrater and Test-Retest Reliability for Toddler Module and Modules 1-4 of ADOS-2*-*PL and Cronbach’s *alphas* for internal consistency analysis.

	Toddler Module		Module 1		Module 2		Module 3	Module 4
	All younger / older with few to no words	Older with some words	Few to no words	Some words	Younger than 5 years	Aged 5 years and older		
**Interrater**								

SA	0.75	0.85	0.89	0.91	0.98	0.86	0.98	0.93
RRB	0.92	0.93	0.94	0.90	0.96	0.97	0.97	0.98
Overall total	0.77	0.83	0.93	0.91	0.97	0.87	0.96	0.94

**Test–retest**								

SA	0.80	0.94	0.93	0.93	0.80	0.74	0.83	0.89
RRB	0.80	0.91	0.82	0.72	0.82	0.41	0.54	0.65
Overall total	0.83	0.95	0.93	0.93	0.87	0.71	0.87	0.88

**Internal consistency**								

SA	0.92	0.86	0.93	0.93	0.85	0.86	0.90	0.92
RRB	0.68	0.64	0.79	0.72	0.79	0.78	0.68	0.76
Overall total	0.92	0.87	0.92	0.93	0.88	0.90	0.89	0.92

*SA, Social Affect; RRB, Restricted and Repetitive Behavior.*

In the case of individual items of ADOS-2-PL, interrater agreement was measured using percent agreement and weighted kappas. Mean percent agreement values for all items in a given module exceeded 92% for all ADOS-2-PL modules. Mean weighted kappas of interrater agreement for items in a given module were equal to or higher than 0.90 for all ADOS-2*-*PL modules, with the exception of Module 4, for which the mean of weighted kappas for items was 0.86.

### Test–Retest Reliability

In order to estimate the stability of measurement over time, the ADOS-2 assessment was conducted twice. **Table 3** shows ICCs calculated for algorithm scales in individual modules of ADOS-2-PL for the test–retest method.

High ICC values (0.71–0.95) were obtained for all algorithms. Only in the RRB domain score was stability lower in three algorithms: in the “Aged 5 years and older” algorithm of Module 2 (0.41), in Module 3 (0.54), and Module 4 (0.65).

### Internal Consistency Reliability

**Table 3** shows the values of Cronbach’s *alpha* coefficients for the SA and RRB domains and Overall Totals in all algorithms of ADOS-2-PL. High internal consistency coefficients were obtained for the Overall Total scores and scores in the SA domain across all ADOS-2-PL algorithms. In the case of the RRB domain in Module T and Module 3, Cronbach’s *alphas* were 0.64 and 0.68, respectively; in the remaining modules they were above 0.70.

### Factor Structure Analysis and Between-Groups Comparisons

In confirmatory factor analysis, Comparative Fit Index (CFI) values between 0.90 and 1.0 are considered representative of a well-fitting model (Byrne, 2009). The second criterion is root-mean-square error approximation (RMSEA), which is satisfactory when below 0.08 (Byrne, 2009). In the case of all analyzed algorithms, including SA and RRB domains and Overall Totals, the RMSEA values were between 0.05 and 0.07. Only in the Toddler Module, in the “Older with some words” algorithm, RMSEA was 0.10. CFI values were within the 0.95–0.97 range, with the exception of the “Older with some words” algorithm in Toddler Module, where CFI = 0.90.

**Table 4** shows the mean values obtained in each ADOS-2*-*PL module in three groups: Autism, Non-autism ASD, and Control, as well as the results of analysis of variance which compared the three groups.

**Table 4:** Mean values in Social Affect (SA) and Repetitive and Restricted Behavior (RRB) domains and Overall Total scores in algorithms of ADOS-2*-*PL and ANOVA results.

Module	Algorithm	Domain	Autism *M (SD)*	Non-autism ASD *M (SD)*	Controls *M (SD)*	*F*	*df*
Toddler Module	All younger/older with few to no words	SA	18.06 (3.98)	15.88 (3.14)	5.48 (5.1)	43.24^∗∗∗^	2.45
		RRB	4.06 (1.25)	3.13 (2.03)	0.91 (1.12)	28.06^∗∗∗^	2.45
		Overall Total	22.12 (4.51)	19 (3.66)	6.39 (5.83)	51.11^∗∗∗^	2.45
	Older with some words	SA	12.00 (6.27)	9.25 (5.62)	4.17 (3.38)	7.91^∗∗^	2.28
		RRB	3.25 (1.89)	3.5 (1.29)	0.87 (1.01)	13.64^∗∗∗^	2.28
		Overall Total	15.25 (7.93)	12.75 (4.35)	5.04 (3.78)	12.21^∗∗∗^	2.28
Module 1	Few to no words	SA	16.72 (3.46)	16.88 (3.18)	5.64 (6.48)	23.44^∗∗∗^	2.34
		RRB	5 (1.61)	2.63 (2.5)	1.73 (1.85)	11.31^∗∗∗^	2.34
		Overall Total	21.72 (4.04)	19.5 (4.9)	7.36 (7.78)	23.84^∗∗∗^	2.34
	Some words	SA	12.83 (3.61)	11.67 (7.64)	3.34 (4.3)	22.37^∗∗∗^	2,41
		RRB	4.5 (1.88)	3.67 (1.15)	1.41 (1.86)	12.70^∗∗∗^	2,41
		Overall Total	17.33 (4.33)	15.33 (8.74)	4.76 (5.53)	24.89^∗∗∗^	2.41
Module 2	Younger than 5 years	SA	10.17 (3.19)	7.67 (2.89)	1.88 (1.98)	40.64^∗∗∗^	2.42
		RRB	4.33 (1.51)	3.6 (2.5)	0.83 (1.2)	16.12^∗∗∗^	2.42
		Overall Total	14.5 (3.39)	11.27 (4.54)	2.71 (2.14)	48.93^∗∗∗^	2.42
	Aged 5 years or older	SA	10.09 (3.11)	6.38 (4.66)	2.25 (2.08)	20.71^∗∗∗^	2.32
		RRB	4.64 (2.38)	3.63 (2.56)	1.31 (1.35)	9.57^∗∗^	2.32
		Overall Total	14.73 (4.98)	10 (6.89)	3.56 (2.25)	20.35^∗∗∗^	2.32
Module 3		SA	12.78 (3.95)	7.52 (3.98)	3.56 (3.93)	35.39^∗∗∗^	2.79
		RRB	3.78 (1.96)	2.9 (2.21)	0.91 (1.11)	23.06^∗∗∗^	2.79
		Overall Total	16.56 (4.95)	10.43 (4.71)	4.47 (4.39)	45.98^∗∗∗^	2.79
Module 4		SA	10.33 (4.69)	8.6 (5.87)	3.38 (4.89)	16.39^∗∗∗^	2.78
		RRB	4.38 (2.55)	3.47 (2.36)	0.81 (1.19)	29.58^∗∗∗^	2.78
		Overall Total	14.71 (6.32)	12.07 (7.00)	4.19 (5.64)	25.31^∗∗∗^	2.78

*M, mean; *SD*, standard deviation; *F*, test statistics; *df*, degrees of freedom, ^∗∗^*p* < 0.01, ^∗∗∗^*p* < 0.001.*

Contrast tests showed that the Autism and Non-autism ASD groups differed significantly in the SA domain in Module 2 (“Younger than 5 years” algorithm, *t* = 2.08, *p* = 0.043) and in Module 3 (*t* = 4.14, *p* = 0.001), in the RRB domain in Module 1 (“Few to no words” algorithm, *t* = 2.95, *p* = 0.006) and in Overall Total scores in Module 3 (*t* = 4.15, *p* = 0.001). Mean score values in the Autism group in the above algorithms were higher than in the Non-autism ASD group. There were no differences between the two groups in the other twenty comparisons. Therefore, comparisons were conducted between the combined Autism and Non-autism ASD (ASD group) and controls. The means and standard deviation of scores in individual scales and algorithms and the results of comparison between the ASD group and the Control group are shown in **Table 5** (means and standard deviations of the Control group are presented in **Table 4**).

**Table 5:** Results of comparisons of the ASD group (Autism and Non-autism ASD) with the Control group.

Module	Algorithm	Domain	ASD group		*t*
			*M*	*SD*	
Toddler Module	All younger/older with few to no words	SA	17.29	3.87	-8.62^∗∗∗^
		RRB	3.79	1.59	-6.65^∗∗∗^
		Overall total	21.08	4.53	-9.30^∗∗∗^
	Older with some words	SA	10.43	6.13	-3.86^∗∗^
		RRB	3.43	1.62	-5.22^∗∗∗^
		Overall total	13.86	6.54	-4.88^∗∗∗^
Module 1	Few to no words	SA	16.77	3.31	-6.69^∗∗∗^
		RRB	4.27	2.18	-3.23^∗∗^
		Overall total	21.04	4.35	-6.46^∗∗∗^
	Some words	SA	12.60	4.34	-5.49^∗∗∗^
		RRB	4.33	1.76	-3.90^∗∗∗^
		Overall total	16.93	5.13	-5.71^∗∗∗^
Module 2	Younger than 5 years	SA	8.38	3.12	-8.97^∗∗∗^
		RRB	3.81	2.25	-6.16^∗∗∗^
		Overall total	12.19	4.43	-10.11^∗∗∗^
	Aged 5 years or older	SA	8.50	4.29	-5.53^∗∗∗^
		RRB	4.33	2.45	-4.10^∗∗∗^
		Overall total	12.83	6.33	-5.73^∗∗∗^
Module 3		SA	9.95	4.73	-8.11^∗∗∗^
		RRB	3.31	2.12	-6.51^∗∗∗^
		Overall total	13.26	5.67	-9.42^∗∗∗^
Module 4		SA	9.67	5.17	-5.20^∗∗∗^
		RRB	4.03	2.49	-7.06^∗∗∗^
		Overall total	13.69	6.63	-6.52^∗∗∗^

*SA, Social Affect; RRB, Restricted and Repetitive Behavior; *M*, mean; *SD*, standard deviation, *t*, Test statistics, ^∗∗∗^*p* < 0.001; ^∗∗^*p* < 0.01.*

Statistically significant differences were found for all comparisons with respect to the SA and RRB domains and Overall Totals. The results of the Control group were lower than the results of the ASD group, suggesting greater severity of ASDs symptoms in that group.

### Sensitivity and Specificity of the Polish Version of ADOS-2

In order to determine the cut-off points and to calculate the sensitivity and specificity of the Polish version of ADOS-2, ROC (Receiver Operating Characteristic) curves were plotted. Since the results of between-groups comparisons suggested that there were no differences between the Autism and Non-autism ASD groups in the majority of comparisons, the groups were combined into one ASD group for the purpose of subsequent analysis. Instead of two cut-offs, as in the original ADOS-2 version (Lord et al., 2012b), only one cut-off was determined for the entire autism spectrum in ADOS-2-PL. The exception was the Toddler Module, which is scored on a three-point ADOS-2 Range of Concern scale. In order to preserve the three-grade structure of the scale, we decided to retain two cut-offs (**Table 6**). When choosing the cut-offs we looked for a value where sensitivity would be at least 80% and specificity as high as possible. Only for the “Older with some words” algorithm in the Toddler Module the sensitivity was 71%. In all of the remaining modules sensitivity was over 90%, with the exception of the “Aged 5 years or older” algorithm in Module 2, in which it was 84%. Specificity in all modules was above 80%, except for the Module 4 and Module 2 “Aged 5 years or older” algorithm, where it was above 70%.

**Table 6:** Sensitivities and specificities of the ADOS-2*-*PL (calculated to differentiate the ASD group and the Control group).

Module	Algorithm	Cut-off^∗^	Sensitivity (%)	Specificity (%)	Positive predictive value	ROC curve (AUC^a^, 95% CI^b^)
Toddler Module	All younger/older with few to no words	14	96	95	0.96	0.98 (0.94-1.00)
		11	96	91	0.92	
	Older with some words	12	71	94	0.83	0.87 (0.71-1.00)
		10	71	88	0.71	
Module 1	Few to no words	13	92	82	0.96	0.99 (0.96-1.00)
	Some words	9	93	83	0.82	0.98 (0.95-1.00)
Module 2	Younger than 5 years	7	91	96	0.95	0.97 (0.94-1.00)
	Aged 5 years or older	6	84	75	0.79	0.91 0.81–1.00)
Module 3		7	90	81	0.83	0.91 0.84–0.98)
Module 4		6	92	74	0.77	0.88 (0.80–0.95)

*^∗^For Modules 1–4 a single cutoff score has been provided; for Toddler Module where results are converted to the three-point Ranges of concern scale two cut-offs has been provided. ^a^Area under the curve. ^b^Confidence intervals.*

### Pair-Wise Agreement between ADOS-2-PL Diagnosis and Clinical Diagnosis, ADI-R and SCQ Scores

**Table 7** presents the results of the analysis of agreement between clinical diagnosis (ASD vs. Non-spectrum), ADI-R and SCQ, and the results for SA, RRB and Overall Total in ADOS-2 modules. Excellent and satisfactory *kappas* were obtained with respect to ADOS-2 and clinical diagnosis in all ADOS-2-PL modules. Similarly, in the case of agreement with the SCQ score, *kappas* were excellent or satisfactory across all modules, with the exception of Module 2, for which the kappa value was fair (0.44). The lowest agreement values were obtained for the consistency between ADOS-2-PL and ADI-R diagnosis. *Kappas* were satisfactory (Module 1) or fair (Modules 3 and 4). The poorest agreement was found for Module 2 (0.35).

**Table 7:** Percent agreement and Cohen’s *kappa* for diagnosis agreement between ADOS-2*-*PL and clinical diagnosis, ADI-R diagnosis and SCQ score.

ADOS-2 Module	Clinical diagnosis	ADI-R diagnosis^∗^	SCQ^∗^
Toddler Module	86.49%, κ = 0.73	—	—
Module 1	92.31%, κ = 0.85	85.71%, κ = 0.72	87.88%, κ = 0.76
Module 2	87.5%, κ = 0.75	67.86%, κ = 0.35	71.70%, κ = 0.44
Module 3	86.42%, κ = 0.73	77.42%, κ = 0.55	83,71%, κ = 0.66
Module 4	82.28%, κ = 0.65	70,37%, κ = 0.40	81,36%, κ = 0.62

*^∗^Participants tested with the Toddler Module were not assessed using ADI-R or SCQ due to their age.*

### Sex, Age and IQ, and Agreement between ADOS-2-PL and Clinical Diagnosis

The relationships between sex and age of participants and their IQ and the level of agreement between diagnosis in ADOS-2-PL and clinical diagnosis was analyzed using logistic regression. No statistically significant relationships were found.

## Discussion

In the present study, we reported the reliability and validity of the Polish version of ADOS-2 (ADOS-2-PL). The ADOS-2-PL is very similar to the original and preserves the structural equivalence, text and format of items, maintains translation accuracy including the contents of items, grammatical structure of questions, difficulty of terminology and lexical similarity of questions. However, to our knowledge, the Polish version is the first one where sounds pronounced by a child (item A1a. “Frequency of Babbling,” ADOS-2 Toddler Module) are not directly translated, but adapted with account being taken of Polish pronunciation and language acquisition and development.

### ADOS-2-PL Reliability

*Kappas* at or above 0.75 were considered excellent, *κ* = 0.60–0.74 were considered satisfactory, *κ* = 0.40–0.59 were considered moderate, and kappas below 0.40 were considered fair (Cicchetti and Sparrow, 1981). Percent agreement between 70% and 79% was considered fair, 80–89% was considered good and above 90% was considered excellent (Cicchetti et al., 1995). Interrater reliability was excellent for all modules, both for Overall Totals and for SA and RRB domains. The same was true of items within individual modules, where Interrater reliability was high also for individual items in all ADOS-2*-*PL modules.

The test–retest correlations for the individual module comparisons indicate excellent stability for the SA domain and Overall Totals for Toddler Module, Modules 1, 3, 4, as well as Module 2 algorithm “Younger than 5 years.” The results for the SA domain and Overall Total of the “Aged 5 years and older” Module 2 algorithm indicate good stability. In the case of the RRB domain, test–retest stability was very good or excellent, except for the “Aged 5 years and older” algorithm in Module 2 (*κ* = 0.41), and the algorithm of Module 3 (*κ* = 0.54) and Module 4 (*κ* = 0.65). In the analyses of the original version of ADOS-2 (Lord et al., 2012b), similarly to ADOS-2*-*PL, RRB domain stability was lower compared to SA domain or Overall Total scores; however, interclass correlations for test-retest reliability were somewhat higher than in the Polish sample, namely 0.68, 0.73 and 0.82 for Modules 1, 2, and 3 respectively. Lower stability in this scale may result from the fact that restricted and repetitive behaviors such as unusual sensory interests, preoccupations, mannerisms or rituals may not be manifested during a 1-h observation for the ADOS-2 protocol. Lower stability in the RRB domain was also found for the modules for which the time from test to retest was relatively long (∼6 months on average) and those used to test older participants who underwent various types of interventions for prolonged periods of time. These factors may have contributed to lower reliability of repeat behavioral measurement in these groups. Nevertheless, the stability of Overall Totals for all ADOS-2*-*PL modules was not affected.

Internal reliability coefficients were very good or excellent for the SA domain and Overall Totals in all ADOS-2 modules (range 0.86–0.93), and satisfactory for the RRB domain (range 0.64–0.79). These findings are similar to those obtained in the original validation study of ADOS (Lord et al., 1999), in which the internal consistency for all modules was slightly lower for the RRB domain totals than for Communication and Social domain totals (the coefficient alpha statistics ranged from 0.47 to 0.65).

To sum up, ADOS-2*-*PL is characterized by high reliability, making it a suitable instrument for individual diagnostics for clinical purposes.

### ADOS-2-PL Validity

Factor analysis confirmed the fit of the two-factor model (with the SA and RRB domains) of the original, revised algorithms (Gotham et al., 2007, 2008) and Toddler Module algorithms (Luyster et al., 2009) to the dataset from the Polish validation sample. In almost all algorithms, CFI values were within 0.95–0.97, while RMSEA 0.05–0.07; these values are considered to indicate good fit (Byrne, 2009). The goodness-of-fit rating was slightly lower only for the “Older with some words” algorithm in the Toddler Module (CFI = 0.90, RMSEA = 0.10). The reason may have been the low subjects-to-item ratio. The same RMSEA value of 0.10 was obtained in the exploratory factor analysis for the “Younger than 5 years” algorithm in Module 2 in the Gotham et al. (2008) study.

Taking into account the goodness of fit indices and the theoretical assumptions underlying the ADOS-2 protocol, we decided to retain the same algorithms in the Polish version consisting of the same items as in the original.

Between-groups comparisons revealed very few differences between participants diagnosed with childhood autism and those with other ASDs. A possible explanation may be the quality of clinical diagnosis, which in Poland is currently formulated without the help of any standardized diagnostic instruments. Another potential contributing factor could be the legal regulations regarding the amount of educational subventions awarded to individuals with these diagnoses, which is several times higher for childhood autism. Such practical concerns may affect the type of clinical diagnosis in individual cases, where professionals wish to ensure that the child is given access to more generous public funding. Clinical diagnoses were not controlled for this type of bias in the present study.

In ADOS-2*-*PL for each of the Modules 1-4 a single cut-off score was provided to differentiate between ASD and Non-ASD groups. The reason was that the comparison of Overall Total scores of participants diagnosed with childhood autism and those diagnosed with other ASD yielded no significant differences (with the exception of Module 3). Additional support for this approach is the current conceptualization of ASDs, which emphasizes quantitative variation in the severity of ASD symptoms in individuals, with the accompanying problems involving clear-cut identification of discrete nosological units within the ASD category (American Psychiatric Association [APA], 2013). A single cut-off was also proposed by Hus and Lord (2014) for ADOS Module 4. This puts limitations on the precise categorization of participants within ASD, which would be welcome in the context of the ICD-10 (World Health Organization [WHO], 2002) diagnostic classification officially adopted in Poland. Since the results of the present study would only allow us to introduce two cut-offs for Module 3, we decided against it in order to maintain a uniform procedure for all modules. In future studies, with more data available, it may be possible to develop precise standardized severity scores that will allow for more precise interpretations of scores. In the Toddler Module, instead of classification cut-off scores we followed the ADOS-2 authors in adopting the “ranges of concern” classification due to the potential instability of early diagnosis (Kleinman et al., 2008; Lord et al., 2012a). All three “ranges of concern” were retained in ADOS-2*-*PL, as in the original; consequently, two cut-offs were preserved in the Toddler Module.

Cut-off scores were selected to achieve the best possible combination of sensitivity and specificity, with particular emphasis on sensitivity. High sensitivity (exceeding 90%) was obtained for all algorithms, with the exception of the Toddler Module Algorithm “Older with some words” (71%) and Module 2 Algorithm “Aged 5 years or older” (84%). The specificity was slightly lower, exceeding 80% for all algorithms, except “Aged 5 years or older” in Module 2 (75%) and Module 4 (74%). For Modules 1–4 of ADOS-2*-*PL, sensitivity was similar to or even higher than in the original ADOS-2 Extended Validation Sample for the comparison between Non-autism ASD and Non-spectrum (Lord et al., 2012b). The specificity of Modules 1-4 of ADOS-2*-*PL compared with the original ADOS-2 Extended Validation Sample for the comparison between Non-autism ASD vs. Non-spectrum was similar or higher in 4 out of 6 algorithms. Lower specificity values were found for Module 4 (74%) and Module 2 for the “Aged 5 years or older” algorithm (75%).

In the case of the ADOS-2*-*PL Toddler Module the sensitivity of the “All younger/older with few to no words” was higher than in the original (Lord et al., 2012a). ADOS-2*-*PL Toddler Module specificity was similar to the values found for the original version, while the sensitivity of the “Older with some words” algorithm was lower than in the original version of ADOS-2 Toddler Module.

The convergent validity for ADOS-2*-*PL was established by comparing ADOS-2*-*PL diagnosis with clinical diagnosis, as well as with the results of ADI-R and SCQ. The comparison yielded excellent and satisfactory agreement for clinical diagnosis and excellent and satisfactory agreement for SCQ score. The exception was Module 2, for which both percent agreement and Cohen’s *kappa* were fair (**Table 7**). We also found lower agreement between ADOS-2*-*PL and ADI-R scores. The lowest agreement with ADI-R was in Module 2. It should be noted that, during our project, data on the psychometric properties of ADI-R and SCQ Polish versions were not available. Therefore, the cut-offs used for both instruments were adopted from their original versions, without prior validation of their suitability.

In general, the reasonably good agreement between ADOS-2*-*PL diagnoses and clinical diagnosis based on a psychiatric evaluation using ICD-10, in combination with other psychometric indices obtained in this study, support the conclusion that ADOS-2*-*PL seems to be suitable for ASD diagnostics.

### Limitations and Strengths

Our findings indicate high reliability and validity of ADOS-2*-*PL, confirming its usefulness both in individual clinical diagnostics of ASDs and in scientific research. The ADOS-2 Polish version has psychometric properties equivalent to those reported for ADOS and ADOS-G foreign language versions (e.g., Bölte and Poustka, 2004; Papanikolaou et al., 2009). As far as we know, the current study is the first evaluation of the reliability and validity of a foreign language version of ADOS-2 with normalization and adaption of pronunciation specific parts. Its findings may serve as inspiration for further investigations of the applications of the ADOS-2 protocol adaptations.

A definite strength of the study was the relatively large validation sample, with over 400 participants in the groups with ASD diagnosis, non-ASD disorders, and developing typically, from toddlers to adults. Furthermore, statistical analyses followed the same procedures as in the original ADOS-2 version, making their psychometric properties readily comparable. Still, the study was not without limitations. Although the total number of participants was relatively high, the groups tested with specific algorithms were rather small. In addition, in the case of a significant proportion of older children and adults diagnosed with ASD, the time from ASD diagnosis was relatively long – more than 1 year, and up to several years for older participants. The results of these individuals may have been affected by therapeutic interventions. Although updated psychiatric opinions regarding the presence and severity of ASD symptoms were available for these participants, since ADOS-2 is an observational protocol assessing the current level of functioning, intervention can obviously significantly affect the subject’s behavioral assessment and the ADOS-2 diagnosis.

Another limitation was that the structure of the sample differed from the demographic structure of the general population in Poland, especially with respect to place of residence, since the majority of participants lived in large cities. Those living in the country made up as little as 8.5% of the sample, which is in stark contrast to the figure for the whole population, i.e., over 39% (Central Statistical Office, 2015) Yearbook.

In addition, with only one cut-off, ADOS-2*-*PL cannot be used to distinguish diagnoses within the autism spectrum. Nevertheless, the study provided valuable information on ADOS-2*-*PL and initiated work on the Polish version of ADOS-2. The work should be continued so that reliable calibrated severity scores can be developed that would make it possible to interpret individual results in the context of scores achieved by other subjects diagnosed with ASD at a similar age and language skills.

## Ethics Statement

This study was carried out in accordance with the recommendations of Faculty’s of Psychology, University of Warsaw Research Ethics Committee with written informed consent from all subjects. All subjects gave written informed consent in accordance with the Declaration of Helsinki. The parents of participating children under 16 years of age signed informed consent forms prior to participation in the study. In the case of participants aged 16 and older, informed consents were signed both by the parent and participant. The protocol was approved by the Faculty’s of Psychology, University of Warsaw Research Ethics Committee. The participants were assessed by examiners experienced in working with persons with disabilities and trained in using all instruments used in the project. All procedures performed in studies involving human participants were in accordance with the ethical standards of the institutional and/or national research committee and with the 1964 Helsinki declaration and its later amendments or comparable ethical standards.

## Author Contributions

Both authors, IC and EP, have had the substantial contribution to the conception and design of the project (IC and EP); translation of the ADOS-2 into Polish (IC); participants recruitment and data collection (IC and EP); data analysis and interpretation of the results (IC and EP); revising the work critically for important intellectual content and adaptation of the ADOS-2 protocols (IC and EP); preparation of the manuscript and approval of the version to be published (IC and EP). Both authors, IC and EP, agree to be accountable for all aspects of the work in ensuring that questions related to the accuracy or integrity of any part of the work are appropriately investigated and resolved.

## Conflict of Interest Statement

IC is an ADOS-2 trainer. The other author declares that the research was conducted in the absence of any commercial or financial relationships that could be construed as a potential conflict of interest.
